# New Mussel Inspired Polydopamine-Like Silica-Based Material for Dye Adsorption

**DOI:** 10.3390/nano10071416

**Published:** 2020-07-20

**Authors:** Marina Massaro, Vincenzo Campisciano, César Viseras Iborra, Leonarda F. Liotta, Manuel Sánchez-Polo, Serena Riela, Michelangelo Gruttadauria

**Affiliations:** 1 Department of Biological, Chemical and Pharmaceutical Sciences and Technologies, University of Palermo Viale delle Scienze, Ed. 17, 90128 Palermo, Italy; marina.massaro@unipa.it (M.M.); vincenzo.campisciano@unipa.it (V.C.); 2 Department of Pharmacy and Pharmaceutical Technology, Faculty of Pharmacy, University of Granada, Campus of Cartuja, 18071 s/n Granada, Spain; cviseras@ugr.es; 3 Andalusian Institute of Earth Sciences, CSIC-UGR, Avenida de las Palmeras 4, 18100 Armilla, Granada, Spain; 4 Istituto per lo Studio dei Materiali Nanostrutturati (ISMN)-CNR, Via Ugo La Malfa 153, 90146 Palermo, Italy; leonardafrancesca.liotta@cnr.it; 5 Department of Inorganic Chemistry, Faculty of Pharmacy, University of Granada, 18071 Granada, Spain; mansanch@ugr.es

**Keywords:** catechol, silica nanoparticles, bioinspired materials, dye adsorption

## Abstract

A straightforward and economic procedure has been developed for the synthesis of a new polydopamine-like silica-based material that has been obtained by oxidation of catechol with KIO_4_ followed by reaction with 3-aminopropyltrimethoxysilane. All techniques adopted for characterization showed that the obtained material is rich in different functional groups and the morphological analyses revealed dimensions in the nanometric range. The hybrid material has been characterized by several techniques showing its polydopamine-like nature, and preliminary observations for dye adsorption have been reported.

## 1. Introduction

The production of smart nanomaterials with specific responses often involves the chemical modification of surfaces with the aim to impart hydrophilic/hydrophobic, antifouling, anti-icing, photo responsive and adhesive properties to the target material.

Catecholamine chemistry, which represents the core of the mussel-inspired coating technology, has been widely exploited for modification and functionalization of surfaces [1], and in this scenario, the surface coating with polydopamine is the most appealing. 

In the last few years, other polydopamine-like materials have been investigated, and the use of dopamine as starting molecule for the formation of the polymeric film coating was replaced by other catechol moiety containing compounds such as tannic acid, caffeic acid, gallic acid, and catechol, among others [2,3]. However, the use of such alternative sources of the catechol moiety require the presence of amine-based derivatives able to undergo co-polymerization processes leading to the formation of the desired polymeric coating film [4,5]. The so-obtained polymers can possess a series of appealing features, such as the case in which catechol was co-polymerized in the presence of polyethylenimine to give rise to a material active in the reduction of water pollution [6]. The crosslinking chemistry of catechol-based molecules with amines was investigated using propylamine and 4-methyl catechol, as model compounds, with the aim to shed some light on the structures of the complex mixture obtained [7]. The use of 3-aminopropyltriethoxysilane (APTES) as amine-derivative has aroused great attention due to its capacity to quickly react with hydroxyl groups present on an hydrophilic surfaces leading to a strong adhesion of the resulting coating layer on different supporting materials [8]. Silvestri et al. reported the polymerization of 5,6-dihydroxyindole-2-carboxylic acid with APTES for targeted subcellular antioxidant protection [9]. Zhou et al. used tannic acid-APTES polymer for different applications including dye adsorption, oil/water separation, and 4-nitrophenol reduction [10], or for enzyme immobilization [3]. Knorr et al. reported the use of polydopamine-APTES hybrids in coating applications [11,12]. Fu et al. described the coating of stainless steel by hydrolytic polycondensation of APTES and polymerization of dopamine [13].

The increased attention to environmental protection has to deal with a world in which large-scale production is required. In this context, textile industry represents one of the most productive sectors that has an inevitable impact on the environment with release of hazardous compounds consisting of organic dyes, such as methyl orange, rhodamine B, among others. It is therefore not surprising that different approaches have been developed for the dye-contaminated waste water treatment. Dyes sorption onto suitable materials represents one of the most advantageous techniques for dye removal due to the high efficiency of the process, energy saving, and easy recycling of sorbent material [14,15,16]. Therefore, it is crucial to find new sorbent materials which can be applied for removal of pollutants.

In the light of the great interest in mussel-inspired chemistry, herein we present a simple and low-cost approach for the synthesis of a polydopamine-like silica-based material by employing the easily available catechol coupled with 3-aminopropyltrimethoxysilane (APTMS) used as source of the amine moiety (PolyCat-Si). We choose catechols as source for the synthesis of polidopamine-like nanomaterials, since it is well known that they play a central role in mussel-mimicking versatility [17].

The obtained hybrid material PolyCat-Si was fully characterized and a possible structure was proposed. In addition, some preliminary results about its potential use for dyes removal were reported. 

## 2. Results and Discussion

**Scheme 1:**
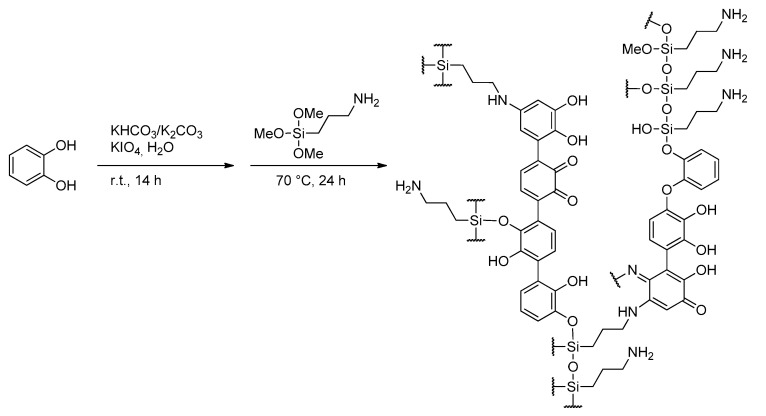
Synthesis of the hybrid material PolyCat-Si. Tentative structure based on characterization data (see discussion).

PolyCat-Si was easily obtained by reaction between catechol (>99%, Sigma-Aldrich, Schnelldorf, Germany) and APTMS (97%, Sigma-Aldrich, Schnelldorf, Germany). In order to form enough *o*-quinone, the oxidant KIO_4_ (99.8%, Sigma-Aldrich, Schnelldorf, Germany) was added and the reaction was carried out at room temperature using carbonate buffer (10 mM, pH 9.0) (K_2_CO_3_ and KHCO_3_ both >99%, Sigma-Aldrich, Schnelldorf, Germany) as solvent. The solution turned very dark in a few minutes and it was left to stir for 14 h. To this dark solution, APTMS was added and the solution was stirred at 70 °C for 24 h. The pH value was chosen slightly lower than the pK_a_ of propylamine (10.71) in order to have a slightly protonated amino group. This approach was chosen with the aim of limiting reactions between the amino group and *o*-quinones since we were interested in the reaction between trimethoxysilane moiety and aryloxy groups. After 24 h, a brown precipitate was formed which was easily filtered, washed, and dried to give the hybrid material PolyCat-Si (Scheme 1). The first step of the synthesis involves the fast oxidation of catechol to o-quinone which can react via reverse dismutation with the starting catechol, to yield highly reactive semi-quinone radicals that can couple to form diaryl compounds. Such compounds can be further transformed/oxidised [18,19]. The mechanism between o-quinone and an amine resembles that of p-quinone and an amine [18] which can be initiated by the Michael-type addition of the amine to a carbon–carbon double bond forming an intermediate that is then isomerized to form aminocathecol which can be subsequently oxidized. This reaction mechanism has been experimentally verified recently by investigating the complex mixture of products obtained by reaction of 4-methyl catechol and propylamine in the presence of NaIO_4_ in Na_2_CO_3_ solution [7]. The main products were formed via Michael-type addition and aryloxyl-phenol coupling, with only a small fraction of products formed via Schiff base reactions [7]. In the present case, in addition to these reactions, hydrolysis and condensation of the trimethoxysilyl moiety can take place. Treatment of 3-aminopropyltrimethoxysilane alone in KHCO_3_/K_2_CO_3_ buffer solution at 70 °C in the absence of the catechol-based oxidized compounds did not give any material clearly showing the role of the latter compounds for the formation of the hybrid material, mainly via Michael-type addition reaction.

The PolyCat-Si hybrid material was characterized by using several techniques confirming the presence of several functional groups as qualitatively reported in Scheme 1. 

**Figure 1:**
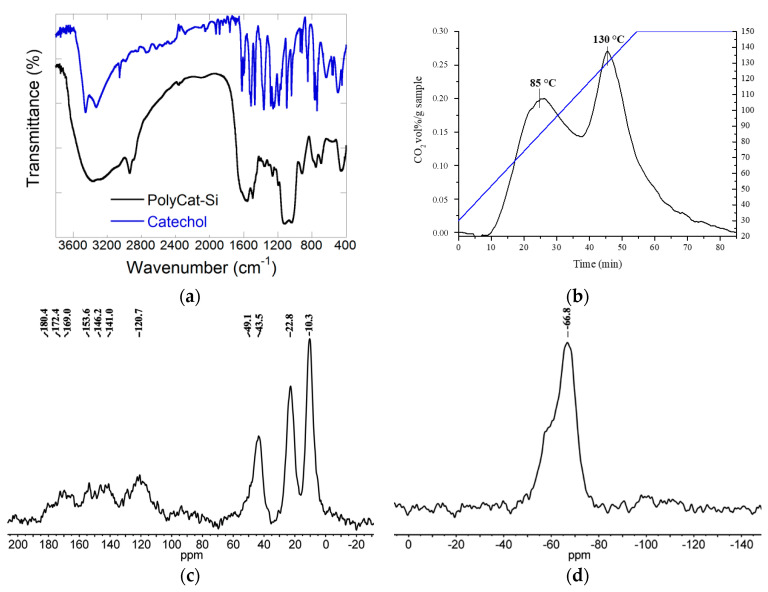
(**a**) FT-IR spectra of catechol and PolyCat-Si; (**b**) Temperature programmed desorption of CO_2_ (TPD-CO_2_) of PolyCat-Si; (**c**) ^13^C MAS NMR spectrum; and (**d**) ^29^Si NMR spectrum of PolyCat-Si.

FT-IR Spectra of catechol and the PolyCat-Si were compared in Figure 1a. As it is possible to observe, the IR spectrum of PolyCat-Si shows relevant differences in comparison to that of catechol confirming the good outcome of the polymerization. In detail, the FT-IR spectrum of PolyCat-Si showed a large and broad peak centered at ca. 3350 cm^−1^ arising from the different stretching vibrations deriving from hydroxyl, amine groups as well as bonded and free water molecules. The peak at ca. 1600 cm^−1^ can be ascribed to the stretching vibration of C=O whereas the C=C stretching vibration of the aromatic rings generated the peak at ca. 1590 cm^−1^. Moreover, the –NH_2_ scissoring vibration mode originates the peak at ca. 1480 cm^−1^. The peaks at 1340 and 1260 were ascribed to the bending vibration modes of –CH_2_ and C–O–H, respectively. Finally, the peaks centered at 1111 cm^−1^ and 1020 cm^−1^ were assigned to the symmetric stretching vibration modes of C–O and to the Si–O–Si stretching vibrations, respectively.

The presence of amino groups of different nature was confirmed by temperature programmed desorption of CO_2_ (TPD-CO_2_) which is a useful tool to estimate the distribution of the basic sites strength and the total basicity (Figure 1b) [20,21,22,23]. The TPD-CO_2_ profile of PolyCat-Si, registered up to 150 °C to avoid any degradation of the material at higher temperature (see below thermogravimetric profile, showed two CO_2_ desorption peaks, at ca. 90 °C and 130 °C, which can be related to different basic groups. Indeed, the occurrence of the two peaks witnesses the presence of CO_2_ retention sites having weak and medium basicity strength as it is related to the desorption temperature of CO_2_ molecules.

To further elucidate the structure of PolyCat-Si we performed some solid-state NMR and XPS characterizations.

^13^C MAS NMR spectrum clearly shows signals related to the aminopropyl chain (Figure 1c). The shoulder at ca. 50 ppm could be due to some residual methoxy groups linked to silicon atom. The very large signals patterns, starting from ca. 110 ppm to ca. 180 ppm are attributable to the complex catechol-based structures, comprising both aromatic catechol rings and *o*-quinones-based structures.

^29^Si NMR spectrum (Figure 1d) shows a signal at -66 ppm due to T3 system RSi(OSi)_3_ that prove the presence of large domains of aminopropylsiloxane moiety and shoulders at ca. -57-60 ppm that could be due to the T2 systems RSi(OMe)–(OSi)_2_, RSi(OPh)–(OSi)_2_, and RSi(OH)–(OSi)_2_ [24].

**Figure 2:**
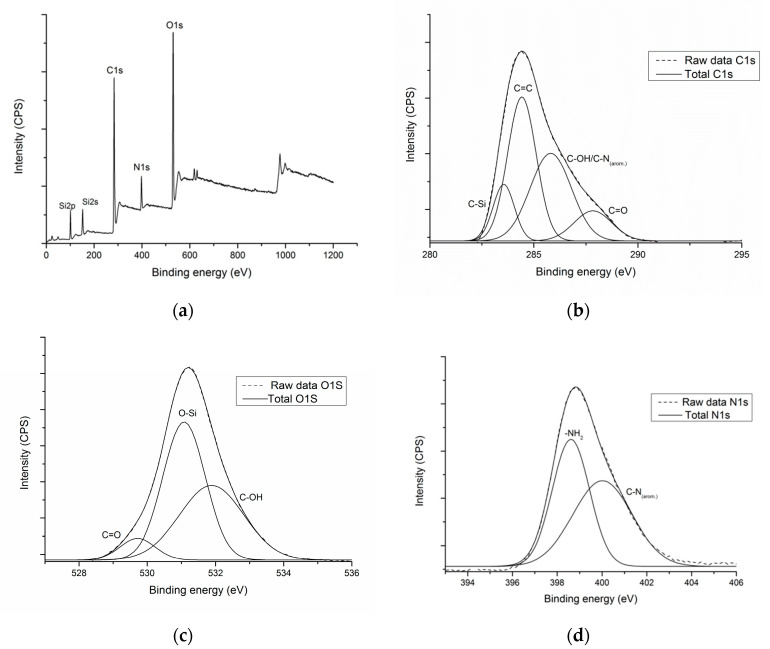
(**a**) XPS survey of PolyCat-Si; (**b**) deconvoluted C 1s spectrum; (**c**) deconvoluted O 1s spectrum; and (**d**) deconvoluted N 1s spectrum.

The XPS survey spectrum for PolyCat-Si and spectra of C1s, N1s and O1s are reported in Figure 2. The hybrid material showed the presence of different functional groups (Table 1 and Table 2). The full-scan XPS spectrum of PolyCat-Si (Figure 2a) revealed the presence of C, N, and O atoms with the corresponding peaks at 284.7 eV (C1s), 399.8 eV (N1s), and 531.7 eV (O1s), respectively. Furthermore, representative peaks for the Si2s and Si2p are also observed, attributing the presence of the silane moieties. The XPS spectrum of C1s (Figure 2b) was deconvoluted into four surface elements corresponding C–Si at 283.6 eV, sp^2^ (C=C) at 284.4 eV, C–OH/C–N_(arom.)_ at 285.8 eV, as well as C=O at 287.8 eV. The O1s spectrum (Figure 2c) also confirmed the coexistence of the aforementioned species (deconvoluted into three peaks at 529.7 eV, 531.1 eV, and 531.9 eV corresponding to C=O, O–Si and C–OH, respectively), as well the N1s spectrum (Figure 2d). The latter was deconvoluted into two peaks centered at 398.6, and 400.1 eV corresponding to –NH_2_ and C–N_(arom.)_, respectively. The C–N aromatic contributions could be ascribed to the presence of amino groups directly linked to the catechol rings, whereas –NH_2_ aliphatic could be related to some unreacted amine tail ends according to ζ-potential results (see infra).

**Figure 3:**
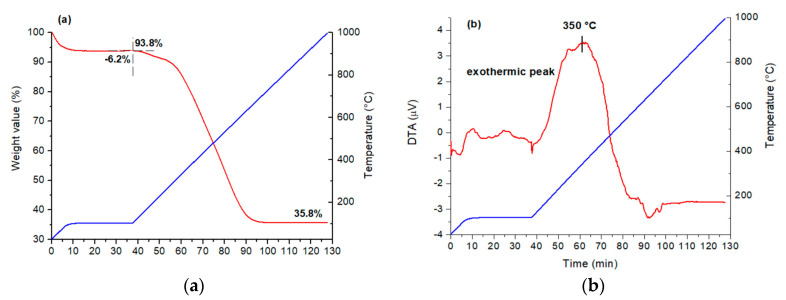
(**a**) Thermogravimetric (TG) and (**b**) Differential thermal analysis (DTA) curves of PolyCat-Si.

In order to study the thermal behavior of PolyCat-Si thermogravimetric analysis was performed. As it is possible to note from Figure 3a,b, PolyCat-Si showed good thermal stability, since the degradation process under air flow started at around 200 °C with light-off temperature between 300–400 °C as confirmed by the exothermic peak centred at 350 °C registered in the differential thermal analysis (DTA) curve. Pre-treatment at 100 °C showed a water content of 6.2%. From the residue, we can argue that 6.36 mmol of APTMS reacted. Considering a complete hydrolysis of the methoxy groups, 1 g of material contains ca. 750 mg of silane and 250 mg of catechol which means a silane:catechol molar ratio of ca. 3. The reaction was carried out with a silane:catechol molar ratio of 2.29. 

Thermogravimetric analysis of other preparations of PolyCat-Si (Figure S1) demonstrated the reproducibility of the synthesis.

Structural characteristics of the hybrid material were also investigated by monitoring its mobility in water through Dynamic Light Scattering (DLS). DLS measurements displayed the presence of a wide population with a hydrodynamic diameter of 411 ± 98 nm. Moreover, ζ-potential measurements showed a negative surface charge (−14.0 ± 1.6 mV) in aqueous solution. By changing the pH of the medium, from 7.0 to 1.0, it was observed an increase in the dimensions of the PolyCat-Si (D_h_ = 1124 ± 168 nm) and a positive surface charge as showed by the ζ-potential value of +30 ± 2 mV. The latter value confirms the presence of terminal –NH_2_ groups which are protonated in acidic medium. Such ζ-potential behavior closely resembles that of polydopamine [25,26,27,28]. 

**Figure 4:**
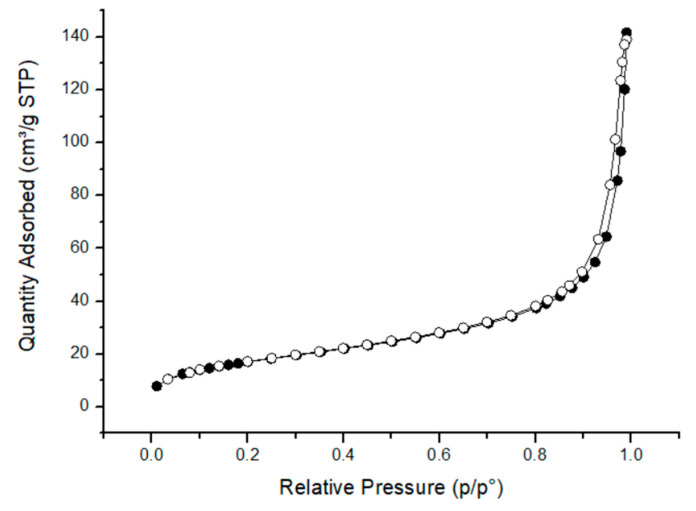
N_2_ adsorption/desorption isotherms of PolyCat-Si registered in the relative pressure range p/p_0_ equal to 0–1.0.

Surface textural properties of the hybrid material were investigated by using N_2_ adsorption/desorption measurements. In particular, the Brunauer–Emmett–Teller (BET) method [23] was applied for the calculation of the specific surface area (SSA); estimation of the pore volumes and pore size distributions was carried out with Barrett–Joyner–Halenda (BJH) method [24] using the desorption isotherm. Figure 4 displays the N_2_ adsorption/desorption isotherms that are of type IV and do not show any evident hysteresis loop except at relatively high pressure 0.9–1.0 p/p^0^. The SSA of the hybrid material was 63 m^2^·g^−1^.

**Figure 5:**
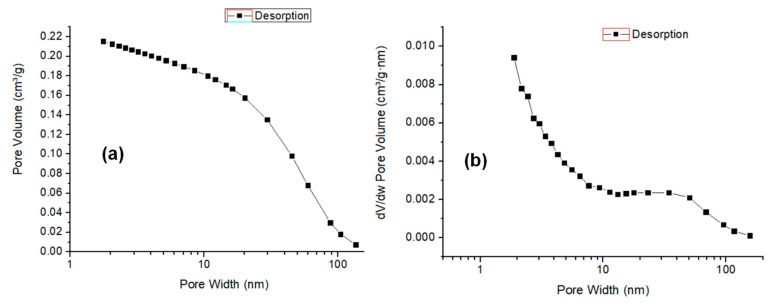
(**a**) Pore volume(cm^3^/g) vs. pore width (nm) and (**b**) dV/dw Pore volume (cm^3^/g·nm) vs. pore width (nm) calculated by Barrett–Joyner–Halenda (BJH) model on the isotherm desorption branch of PolyCat-Si.

The pore size distribution reported in Figure 5a,b indicates a wide distribution of pores in the entire range, 2–100 nm, with the main contribution of mesopores between 2–10 nm. The average pore size calculated by BJH model was equal to 7.4 nm with a cumulative volume of pores of 0.21 cm^3^·g^−1^.

**Figure 6:**
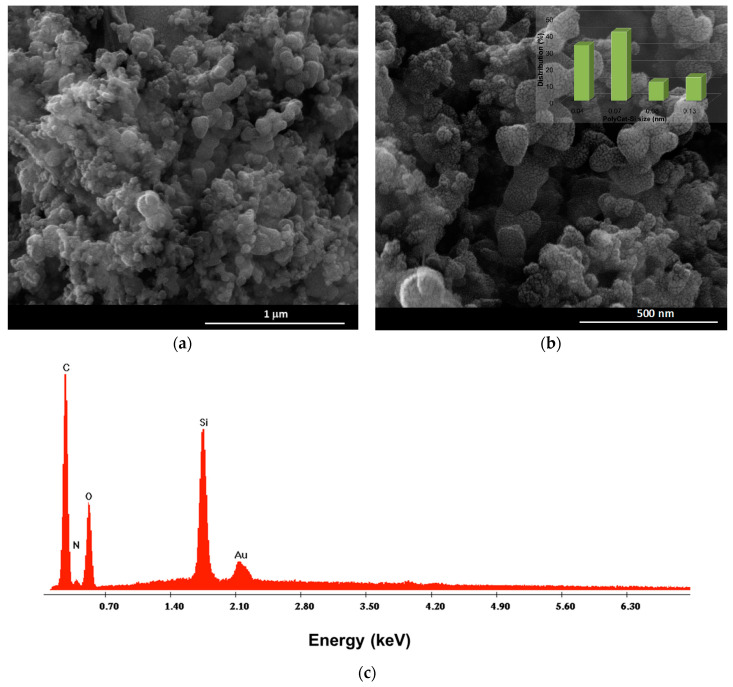
(**a**,**b**) SEM images of PolyCat-Si nanomaterial. The inset shows the PolyCat-Si size distribution (n = 50); (**c**) EDS pattern. Sample is coated with gold.

SEM investigations of PolyCat-Si (Figure 6) showed regular round-shaped nanostructures, typical of polidopamine-like materials, with dimensions in the nanometric range (ca. 70 nm). In addition, EDS analysis showed the signals of silicon, oxygen, carbon, and nitrogen which further confirm the presence of these elements in the PolyCat-Si.

**Figure 7:**
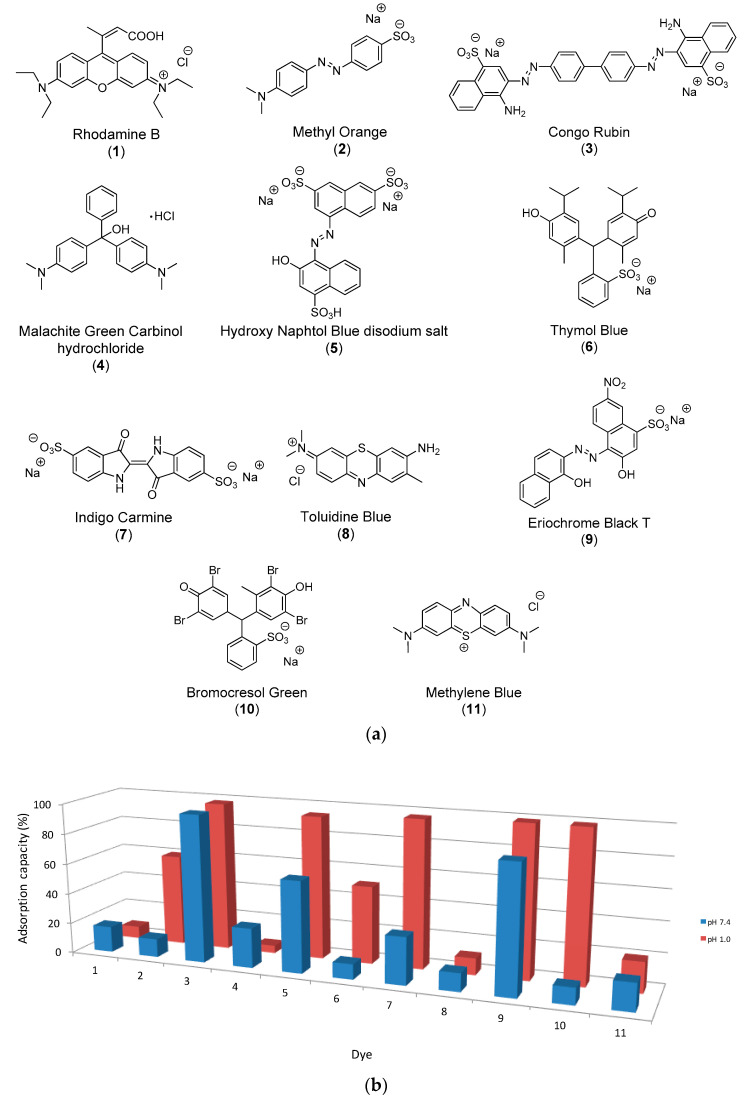

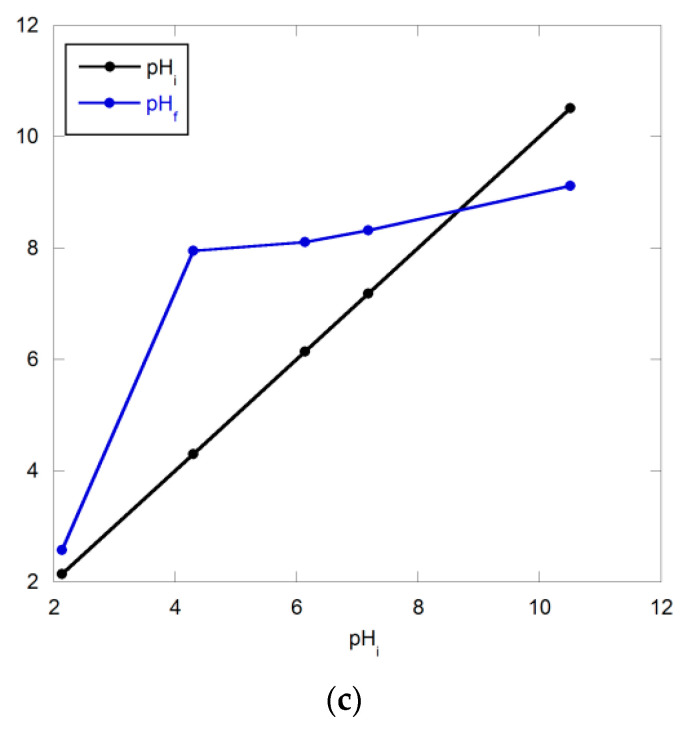
(**a**) Structure of the investigated anionic and cationic dyes; (**b**) adsorption capacities of PolyCat-Si for the investigated dyes at pH 1.0 and 7.4; (**c**) determination of pH_PZC_ for the PolyCat-Si.

In order to assess the possible use of PolyCat-Si as potential system for dyes removal, adsorption experiments were performed. Therefore, we determined the adsorption capacity of PolyCat-Si for 11 anionic and cationic dyes (Figure 7a) at different pH values, namely, 1.0 and 7.4. The results showed that the PolyCat-Si was able to efficiently absorb anionic dyes, at both the pH values investigated. However, the acidic medium allowed for a better adsorption capacity of PolyCat-Si (Figure 7b). This behavior was explained taking into account the pH_pzc_ of the nanomaterial. The determination of this value is crucial to highlight some alterations in the protonation–deprotonation characteristics of the surface of a material since it represents the point at which the surface acidic (or basic) functional groups no longer contribute to the pH value of the solution [29]. We found that the PolyCat-Si material possesses a pH_PZC_ of ca. 8.7 (Figure 7c). Therefore, its surface in pHs lower than pH_PZC_ shows a net positive charge, and thus, the nanomaterial could attract by electrostatic interactions the anionic dyes. However, most of the dyes investigated present pK_a_ values in the pH range between 3 and 6, so by increasing the pH (from 1.0 to 7.4), the dyes turn into their neutral form and the attractive forces decrease explaining the slight adsorption ability decrease at neutral pH.

**Figure 8:**
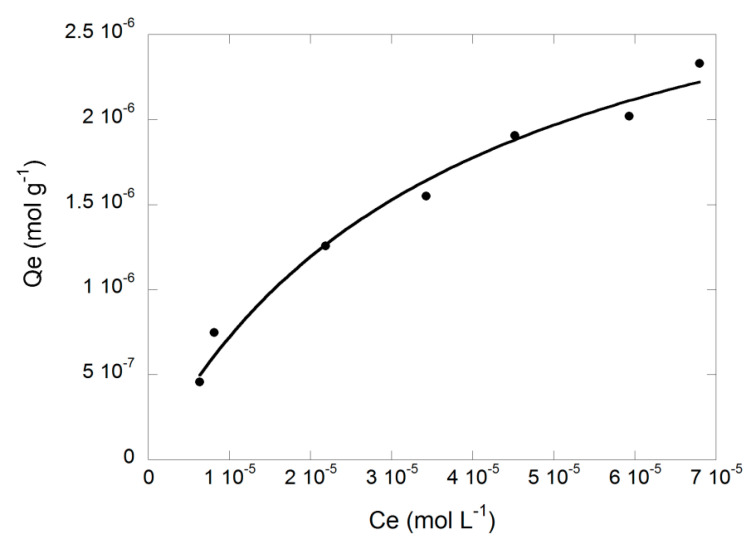
Adsorption isotherm of methyl orange (MO) on the hybrid material in HCl 0.1 N (results correspond to the mean ± standard error mean of three independent assays).

Adsorption isotherm experiments were also carried out to evaluate the adsorption capacity of PolyCat-Si by choosing methyl orange (MO) as the dye model at pH 1.0 (HCl 0.1 N). In Figure 8, the plot of the equilibrium amount of dye adsorbed into the hybrid material (Qe, mol·g^−1^) vs. the equilibrium dye concentration in solution (Ce, mol·L^−1^) was reported. Experimental data were analyzed by the using of the Langmuir and the Freundlich models. It was found that the adsorption data are only well fitted by the Langmuir equation [Q_m_ = (3.4 ± 0.3) × 10^−6^ mol·g^−1^, K_L_ = (2.6 ± 0.5) × 10^5^ L·mol^−1^], indicating that the adsorption follows the Langmuir adsorption isotherm, suggesting homogeneous nanocomposite and monomolecular layer adsorption.

To gain further insight into the adsorption mechanisms, the kinetics of methyl orange adsorption onto PolyCat-Si was also investigated (Figure S2). It was found the dye adsorption is a slow process and the equilibrium was reached only after 800 min. The kinetic data were fitted by the Weber–Morris model and by a first-order kinetic model [1]. It was found that the kinetic data are better analyzed by a Weber–Morris model (*k* = 6.1 ± 0.2 min^−1^; *R*^2^ = 0.9857) indicating that the adsorption is ruled by an intra-particle diffusion.

The organic dye adsorbed onto the material can be easily removed by washing the PolyCat-Si with methanol, and therefore the material could be re-used.

It could be noteworthy to compare the adsorption performances of the PolyCat-Si towards anionic dyes (such as methyl orange, congo red, and so on) and those of other adsorbent materials already present in literature. PolyCat-Si in the best experimental conditions showed an adsorption capacity of 24 mg·g^−1^ towards MO.

It should be noted that although our material possesses an adsorption capacity comparable with that of other polydopamine-like materials (Table 3), it possesses the great advantage to have free amino groups, which are available for further modifications which could improve the adsorption capacity of a post-modified PolyCat-Si material.

## 3. Conclusions

In conclusion, new mussel inspired polydopamine-like silica-based material based on catechol and APTS (PolyCat-Si) was successful synthetized and characterized by several techniques that highlighted the presence of several functional groups (FT-IR, solid state NMR, EDX, TPD-CO_2_, and XPS). Furthermore, the textural properties, the thermal stability and mobility in aqueous regime were also investigated (BET, TGA, DLS, and ζ-potential measurements). The morphology of the PolyCat-Si was imaged by SEM investigations. In addition, it was found that PolyCat-Si presented dimensions in the nanometric range. The potential feasibility of the PolyCat-Si hybrid material as a decontaminant of wastewater was investigated by studying its adsorption ability towards 11 anionic and cationic organic dyes. The ionicity of the material and the pK_a_ values of the dyes were taken into account to explain the adsorption capacity of PolyCat-Si. The obtained results showed a promising application of the PolyCat-Si hybrid material in the bioremediation field.

## Figures and Tables

**Table 1:** Percentage of Si, C, N, and O atoms, as determined by XPS measurements.

Atom	%
Si	7.3
C	61.4
N	7.4
O	23.6

**Table 2:** Functional groups present in the PolyCat-Si obtained by the deconvolution of XPS spectra.

Functional Groups	Abundance (%) ^a^
**C**	
C–Si	12
C=C	41
C–OH/C–N_(arom.)_	36
C=O	11
**N**	
NH_2_	48
C–N_(arom.)_	52
**O**	
C=O	7
O–Si	51
C–OH	42

^a^ The percentages refer to the atom in bold in each functional group.

**Table 3:** Comparison of the adsorption capacity of other adsorbents towards MO in the previous studies at 298 K.

Material	Q_e_ mg·g^−1^	Ref.
PDA-GO	30	[30]
Poly(catechol-tetraethylenepentamine-cyanuric chloride)@hydrocellulose (PCEC-C)	37.2	[31]
PCL/PEO@PDA-45	60.2	[32]
Fe_3_O_4_@PDA NPs	2.1	[33]
PDA microspheres	almost zero	[34]
PolyCat-Si	24	this work

## Supplementary Materials

The following are available online at https://www.mdpi.com/2079-4991/10/7/1416/s1. Experimental details Figure S1: Thermogravimetric curve of PolyCat-Si (2^nd^ preparation as example), Figure S2: Kinetic adsorption of MO on PolyCat-Si in HCl 0.1 N.
