# Supplementary material for: New Mussel Inspired Polydopamine-Like Silica-Based Material for Dye Adsorption
Source: Nanomaterials (Basel). 2020 Jul 20;10(7):1416. doi: 10.3390/nano10071416 (PMC7408388; doi:10.3390/nano10071416)
Supplement: Supplementary file 1 [file nanomaterials-10-01416-s001.pdf]

New mussel inspired polydopamine-like silica-based material for dye adsorption

Marina Massaro,[1#] Vincenzo Campisciano,[1#] César Viseras Iborra,[2,3] Leonarda F. Liotta,[4] Manuel Sánchez-Polo,[5] Serena Riela,[1*] Michelangelo Gruttadauria[1*]

[1] Department of Biological, Chemical and Pharmaceutical Sciences and Technologies, University of Palermo Viale delle Scienze, Ed. 17 90128, Palermo, Italy. marina.massaro@unipa.it (M.M.), vincenzo.campisciano@unipa.it (V.C.), serena.riela@unipa.it (S.R.), michelangelo.gruttadauria@unipa.it (M.G.)

[2] Department of Pharmacy and Pharmaceutical Technology, Faculty of Pharmacy. University of Granada, Campus of Cartuja, 18071 s/n, Granada, Spain. cviseras@ugr.es (C.V.I.)

[3] Andalusian Institute of Earth Sciences, CSIC-UGR. Avenida de las Palmeras 4, 18100 Armilla, Granada, Spain.

[4] Istituto per lo Studio dei Materiali Nanostrutturati (ISMN)-CNR, Via Ugo La Malfa 153, 90146 Palermo, Italy. leonardafrancesca.liotta@cnr.it (L.F.L.)

[5] Department of Inorganic Chemistry, Faculty of Pharmacy, University of Granada, 18071 Granada, Spain. mansanch@ugr.es (M.S.-P.).

* Correspondence: serena.riela@unipa.it Tel.: +39 091 23897546 (S.R.); michelangelo.gruttadauria@unipa.it Tel.: +39 091 23897534 (M.G.).

Total number of pages: 9
Total number of Figures: 2

Content

1. Experimental details……………………………………………………...………………....S3
2. Thermogravimetric curve of PolyCat-Si..S6
3. Kinetic adsorption of MO on PolyCat-Si in HCl 0.1 N...S7
4. References..S8

EXPERIMENTAL

All chemicals were obtained from Sigma-Aldrich and used as received.

The thermogravimetric analysis (TGA) of the material was performed in a TGA/DSC1 STAR System from Mettler Toledo Inc. The sample (15 mg) was subjected to a pre-treatment in air flow (30 mL/min) from 25 °C to 100 °C with a heating rate of 10 °C/min and holding time at 100 °C for 30 min, in order to remove physisorbed water. Then, the temperature was increased from 100 to 1000 °C under air flow (30 mL/min) and the weight loss occurring during this step was considered in order to calculate the organic weight content of the silica-based material.

The basicity of the sample was investigated through CO_2 temperature-programmed desorption (CO_2-TPD) carried out in a Micromeritics Autochem 2950HP apparatus equipped with a thermal conductivity detector (TCD) and an IR analyzer (ABB Uras 14). The sample (200 mg) was put in a quartz U shaped reactor with an inner diameter of 12 mm, electrically heated in a furnace. The adsorption of CO_2 was performed at 30 °C under pure CO_2 (50 mL/min) for 1 h, then He (50 mL/min) was used to remove the physically adsorbed CO_2 purging for 1 h at room temperature. Finally, the temperature-programmed desorption procedure was carried out under He flow (50 mL/min) from 30 °C to 150 °C (heating rate of 2 °C/min), holding time at 150 °C for 30 min.

N_2 adsorption–desorption measurements were carried out at 77 K by using a volumetric adsorption analyzer (Micromeritics ASAP2020). The samples were preliminarily subjected to a pre-treatment at 50 °C under reduced pressure (0.1 mbar) for 1 h. The specific surface area was calculated applying the BET method in the p/p_0 = 0.05-0.30 range, whereas the estimation of the pore volume and average pore size were made applying BJH method to the desorption branch of the isotherm. Values obtained are affected by an incertitude of ±10%.

A Bruker Avance II 400 MHz spectrometer was used to acquire ^{13}C and ^{28}Si CPMAS NMR CPMAS NMR spectra applying a MAS speed of 8 kHz, 1024 scans, a contact time of 1.5 ms, a delay time of 3 s, and an excitation pulse of 5.4 µs on the 1H nucleus.

Dynamic light scattering (DLS) and ζ-potential measurements were performed by using a Zetasizer Nano-ZS (Malvern Instruments) apparatus. The measurements were carried out on aqueous dispersions (10^{-3} wt%) at 25.0 ± 0.1 °C. For DLS investigations, a scattering angle of 173° and a wavelength of 632.8 nm were set. The obtained field-time autocorrelation functions were analyzed by Inverse Laplace Transformation.

FT-IR spectra (KBr) were acquired with an Agilent Technologies Cary 630 FT-IR spectrometer.

XPS measurements were performed using an ESCA 5701 from Physical Electronics equipped with MgK X-ray source (hν = 1253.6 eV) and hemispherical electron analyzer. Survey and multi-region spectra were recorded at C1s, O1s, and N1s, photoelectron peak.

UV–vis spectra were acquired by means of Beckmann DU 650 spectrometer.

The morphology of the PolyCat-Si was studied using an ESEM FEI QUANTA 200F microscope with EDX probe. The measurement was carried out in high-vacuum mode (<6 × 10^{-4} Pa). The energy of the beam was 20 keV, and the working distance was 10 mm. Minimal electron dose conditions were employed to avoid damaging the sample. Before each experiment, the sample was coated with gold in argon (60 s) by means of an Edwards Sputter Coater S150A to avoid charging under an electron beam.

The pH of point of zero charge of PolyCat-Si, pH_{PZC}, was determined as follows [1]: The pH of a series of 20 mL 0.01 M NaCl solutions was adjusted to a value between 2 and 10 by adding HCl 0.1 M or NaOH 0.1 M solution in closed Erlenmeyer flasks. Before adjusting the pH, all solutions were degassed by purging Ar gas to remove dissolved CO_2. The pH of these solutions was recorded as the initial pHs (pH_i). Then, 0.1 g of PolyCat-Si was added and the final pH (pH_f) was measured after 24 h. Finally, the plots of pH_f versus pH_i and also pH_i vs. pH_i were constructed which the intersection of these curves determines the pH_{PZC}.

Synthesis of PolyCat-Si

To a buffer solution ($KHCO_3/K_2CO_3$, pH 9, 10 mM, 100 mL) was added catechol (480 mg, 4.36 mmol) and KIO_4 (500 mg, 2.17 mmol). In a few minutes, the solution became very dark, and it was stirred at room temperature overnight (14 h). After this period, 3-aminopropyltrimethoxysilane (1.79 mL, 10 mmol) was added and the solution stirred for 24 h at 70 °C. After cooling at room temperature, the solution was filtered and the precipitate was washed with water, ethanol, and diethyl ether. The solid was dried at 60 °C under reduced pressure (1.38 g).

Batch adsorption experiments

A total of 5.0 mg of PolyCat-Si were weighed in sealed vessels and 2 mL of dye solution (1 × 10^{-4} M with the exception of dyes 1, 2, and 11 used with a concentration of 1 × 10^{-5} M) in different buffered solutions (namely HCl 0.1 N and phosphate buffer pH 7.4) were added at 25 °C. After sonication, the obtained dispersions were vortexed and left to stir overnight. The mixtures were centrifuged and the supernatants were collected and analyzed by UV–vis spectrophotometer at the maximum absorption wavelength for each dye. The removal efficiency (RE (%)) was calculated by:

$$RE\ (\%) = \frac{(C_0 - C_e)}{C_0} \times 100 \qquad \text{(Eq. 1)}$$

where C_0 and C_e are initial and equilibrium concentrations of the dyes (M), respectively.

Adsorption isotherm

Then, 2.5 mg of PolyCat-Si were weighed in sealed vessels and 1 mL of a MO solution (concentration ranging between 1 × 10^{-5} - 2.5 × 10^{-4} M) in HCl 0.1 N were added. The obtained dispersions were left to stir for 12 h at 25 °C to reach equilibrium. The equilibrium adsorption capacity Q_e (mol g^{-1}) was calculated by the following equation:

$$Q_e = \frac{(C_0 - C_e) \cdot V}{M} \qquad \text{(Eq. 2)}$$

where C_0 and C_e are initial and equilibrium concentrations of MO (M), respectively, M is the weight of PolyCat-Si (g), and V is the volume of MO solution (L).

The adsorption data were analyzed by the Langmuir (Eq. 3) and Freundlich (Eq. 4) models expressed as follows:

$$Q_e = \frac{KQ_m C_e}{1 + KC_e} \qquad \text{(Eq. 3)}$$

$$Q_e = K_F C_e^{1/n} \qquad \text{(Eq. 4)}$$

where Q_e (expressed as mol g^{-1} and mg g^{-1}, for Langmuir and Freundlich models, respectively) is the amount of adsorbed dye at equilibrium; C_e (expressed as mol L^{-1} and mg L^{-1}, for Langmuir and Freundlich models, respectively) is the concentration of dye in solution at equilibrium; Q_m (mol g^{-1}) is a constant standing for the maximum monolayer adsorption capacity of adsorbent. K_L is a constant corresponding to the affinity of adsorbent towards adsorbate, K_F is a Freundlich constant related to adsorption capacity (L g^{-1}). The other Freundlich constant *n* is a measure of the deviation from linearity of the adsorption and used to verify types of adsorption.

Adsorption kinetic

The batch experiments were carried out for investigating the adsorption kinetics of MO onto PolyCat-Si at room temperature in HCl (0.1 M) pH 1. For all experiments, PolyCat-Si (5 mg) were added into 2 mL solution of MO (5× 10^{-5} M). The amount of MO adsorbed at time *t* (Q_t, mol g^{-1}) was calculated by the following equation:

$$Q_t = \frac{(C_0 - C_e) \times V}{M} \qquad \text{(Eq. 5)}$$

where C_0 and C_e are initial and equilibrium concentrations of MO (M), respectively, M is the weight of PolyCat-Si (g) and V is the volume of MO solution (L).

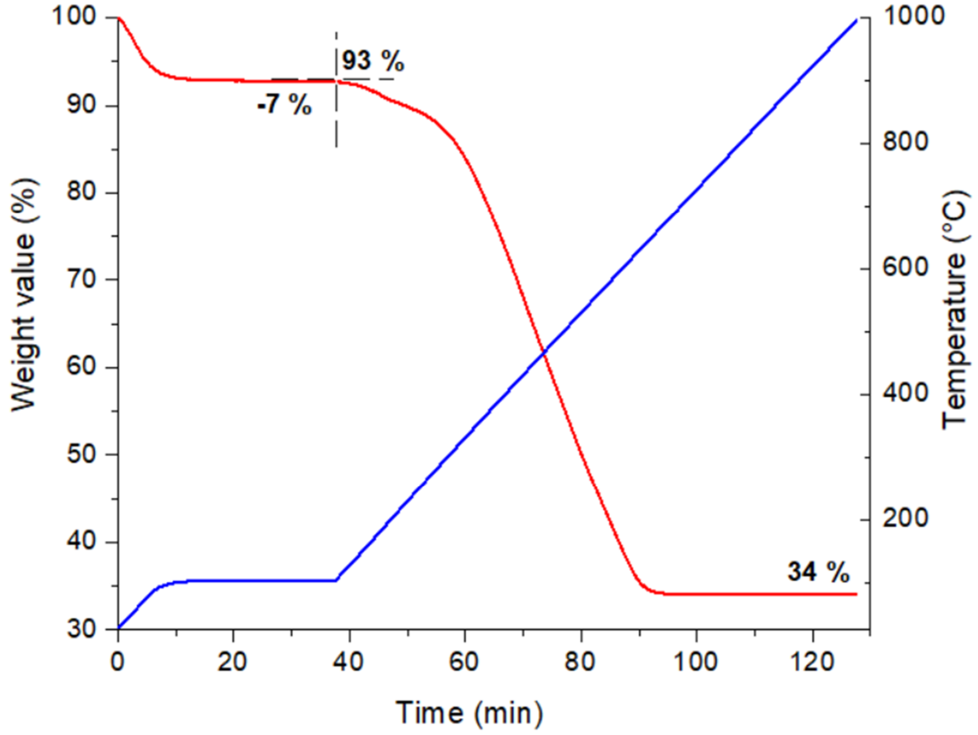

Figure S1. Thermogravimetric curve of PolyCat-Si (2nd preparation as example).

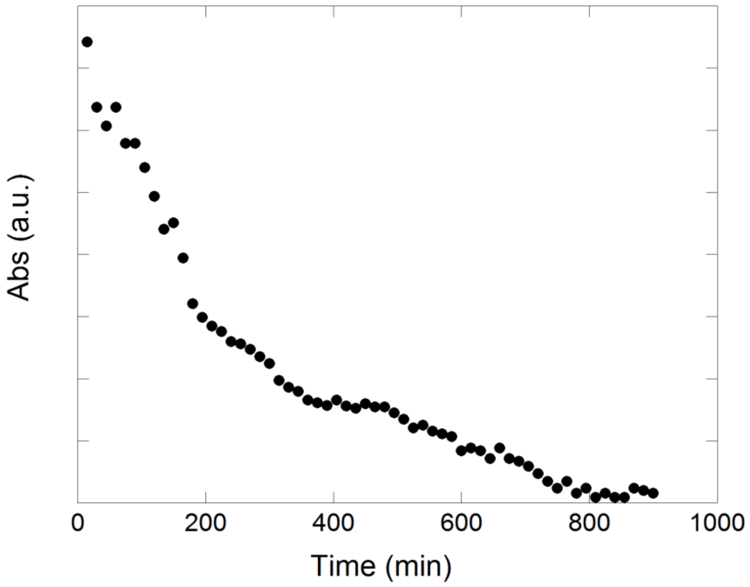

Figure S2. Kinetic adsorption of MO on PolyCat-Si in HCl 0.1 N.

References

1. Nezamzadeh-Ejhieh, A.; Zabihi-Mobarakeh, H. Heterogeneous photodecolorization of mixture of methylene blue and bromophenol blue using CuO-nano-clinoptilolite. *Journal of Industrial and Engineering Chemistry* **2014**, *20*, 1421-1431, doi:https://doi.org/10.1016/j.jiec.2013.07.027.
